# Low defect and high electrical conductivity of graphene through plasma graphene healing treatment monitored with in situ optical emission spectroscopy

**DOI:** 10.1038/s41598-021-99421-7

**Published:** 2021-10-13

**Authors:** Mohammad Salehi, Parnia Bastani, Loghman Jamilpanah, Abbas Madani, Seyed Majid Mohseni, Babak Shokri

**Affiliations:** 1 Laser and Plasma Research Institute, Shahid Beheshti University, 19839 Tehran, Iran; 2 Department of Physics, Shahid Beheshti University, 19839 Tehran, Iran; 3 AMO GmbH (Advanced Microelectronic Center), Aachen, Germany; 4 Department of Engineering, The University of Cambridge, Cambridge, UK

**Keywords:** Materials science, Nanoscience and technology

## Abstract

Fundamental studies on graphene (Gr) and its real device applications have been affected by unavoidable defects and impurities which are usually present in synthesized Gr. Therefore, post treatment methods on Gr have been an important subject of research followed by the community. Here, we demonstrate a post-treatment of cm-sized CVD-grown graphene in a Radio Frequency-generated low-pressure plasma of methane and hydrogen to remove oxygen functional groups and heal the structural defects. The optimum plasma treatment parameters, such as pressure, plasma power, and the ratio of the gases, are optimized using in-situ optical emission spectroscopy. This way we present an optimal healing condition monitored with in situ OES. A twofold increase in the conductivity of plasma-treated Gr samples was obtained. Plasma treatment conditions give insights into the possible underlying mechanisms, and the method presents an effective way to obtain improved Gr quality.

## Introduction

Preparing high-quality CVD-grown graphene (Gr) layers to be implemented in electronic devices has been a great challenge, in both growth and transferring steps. Some examples of undesired effects in Gr after the growth and transferring steps are usually defects, corrugation, cracks, metal and polymer residuals, oxygen functional groups, etc^1–4^. Such unlikely damages have limited fabrication technology and large-scale production ability of Gr for widespread and scalable applications like, for example, electronics, energy conversion and storage, chemical and biological sensing, catalysis, or biomedicine hitherto^5–12^.

As no replacement is yet present for the Gr layer's scalable production otherwise than the current CVD technology and follows up transferring process, different approaches have been suggested to improve the transferred Gr layer's quality based on some alternative treatments.

A critical aspect of current transfer methods that should be resolved is polymer residuals. Various methods, including current-induced cleaning^13^ and annealing at high temperatures in ultrahigh vacuum or Ar/H_2_ or N_2_/H_2_ environment^14,15^ have been used to remove polymer residues. In addition to the difficulty of the process and the need for high temperatures, these methods create defects and disorders in Gr^16^. Hence, new non-destructive methods are demanded to remove the polymer residues and eliminate the defects formed within the transfer process.

Plasma treatment is an efficient method for Gr post-processing and defect engineering^17,18^, and also provides a promising technique for Gr surface cleaning and healing for a high surface area with a fast processing time^19–22^. However, the exploration of plasma processing is still an open field to be developed for further functionality. Different gases like Hydrogen, Argon, and Nitrogen have been used to eliminate polymer residues from the surface of Gr^20,21^. Although Hydrogen plasma can clean polymer contaminations, defects in the Gr are produced through chemical and physical processes^19,23,24^. Using a mixture of Hydrogen and Methane gases within various steps has shown low defect density and better removal of polymers. Four different gas combinations of hydrogen and methane were studied in previous studies to obtain a proper method^16^. A microwave plasma source has been used to generate hydrogen-methane (30/20 sccm) mixed plasma, which had the pressure of 5.8 Torr and the power of 300 W in the whole treatment processes (30 s). The results of that study showed that the simultaneous use of hydrogen and methane as plasma gas would have better results by controlling the surface reactions during the plasma treatment and achieving a nearly perfect and residual free Gr. There is also another study which used the same mixture of methane and hydrogen plasma at 700–900 °C at much lower pressure (0.2–0.4 Torr) and longer treatment duration (30 min)^25^. However, based on the aforementioned studies that used plasma treatment, no in-situ analyses were made to investigate the exact mechanism of Gr healing process. Thus, to accomplish the mechanism of plasma treatment, in this work, we use in-situ optical emission spectroscopy (OES) to reveal the exact composition of plasma, and monitor the plasma parameter and effective plasma components in the graphene healing and polymer residual removal process. OES is an ideal method to measure the intensity of light emitted from the plasma to detect plasma components in hostile plasma reaction environments. Its versatility is evident as it can be applied for any research that involves the formation of plasma. Spectroscopy is a non-invasive and non-destructive method that does not affect plasma and material processing^26–28^. We introduce a method that obliterates polymer residues, reduces the number of Gr layers, and eliminates defects and oxygen functional groups on the surface of Gr without damaging it by using a mixture of methane and hydrogen plasma without using high temperature treatment. Also, the treated sample is shown to have enhanced electrical transport properties. Results can be considered for obtaining better electronic devices through plasma treatment and illuminate the deriving mechanisms behind plasma treatment of Gr.

## Methods and materials

Gr was synthesized using CVD, on highly pure Cu foil (20 µm thickness) substrate as a catalyst, and by using acetylene gas (C_2_H_2_) as the carbon source. Usage of C_2_H_2_ for Gr growth and CH_4_ for Gr healing experiments is due to several reasons, e.g., Acetylene's p-bond allows it to adhere firmly to transition metals. It facilitates the dissociation of the hydrocarbon for the formation of graphene sheets^29^. A high pyrolysis rate in Acetylene also enables it to significantly decrease the defects in graphene, which is due to the divacancy defects healing mechanism^30^. In addition, the use of Acetylene has been reported to reduce growth temperature^31^. The synthesized Gr was transferred on Si/SiO_2_ substrate by wet transfer method using PMMA polymer and FeCl_3_ (1 M) etchant. The Gr healing and polymer residual removing process was carried out via a mixture of methane and hydrogen plasma (30 sccm of CH_4_ and 45 sccm of H_2_) using a low-pressure RF capacitively coupled plasma reactor (RF-CCP). The gas pressure and RF plasma power were 80 mTorr and 80 W, respectively. To characterize the plasma generated in the chamber, OES was performed for different conditions such as gas ratio (CH_4_/H_2_), different powers (in the range of 10–200 watts), and pressures between 40 and 200 mTorr. The spectrum of plasma generated in a low-pressure chamber is transferred to the spectrometer (AvaSpec 3648, Avantes) by an optical fiber placed in front of a quartz window embedded inside the chamber's body to collect light from areas of plasma where the sample was placed. The sample's topographical data was acquired by atomic force microscopy (AFM) in non-contact mode (EasyScan 2, Nanosurf) under ambient conditions. Raman spectroscopy was carried out with a Teksan Raman microscope spectrometer (Takram P50C0R10, $\lambda$ = 532 nm). The conductivity test of Gr was performed using a Keithley 2450 source meter in a two-probe mode.

## Results and discussion

In the plasma medium, the decomposition of CH_4_ occurs mainly by the electron impact and dehydrogenation process and forming active methane species (CH_x_). To determine the repairing performances during the CH_4_ plasma treatment, the density functional theory (DFT) calculations have been carried out in previous studies^25^. One of the most likely methane radicals to form in plasma is CH_2_, which initially forms a bond with two carbon atoms in the vacancy region and can repair the defect^25^. Hydrogen atoms released from this process can also form molecular hydrogen considering the barrier energy of the reaction between the vacancy and other possible species in the chamber. It can be concluded that the CH and CH_2_ radicals are dominant in the repairing process due to the lower barrier energy. The amount of hydrogen generated in this process is not enough to perform the expected hydrogen plasma roles, so we have to add pure H_2_ to the chamber. Hydrogen plays a vital role in controlling the structure, domains, and layers and removing oxidizing agents^25^. Therefore, methane with a ratio of hydrogen to the carbon of 4 to 1 is more suitable in this process^16,25,32^. Acetylene plasma products include species with an even number of carbon atoms, while the process of filling vacancies is done by active single carbon species, especially CH and CH_2_^32,33.^ Production of dust particles is another drawback of using Acetylene^34^. The balance between etching and Gr growth can be adjusted through various parameters such as gas ratio, pressure, and plasma power^16,25^.

**Figure 1:**
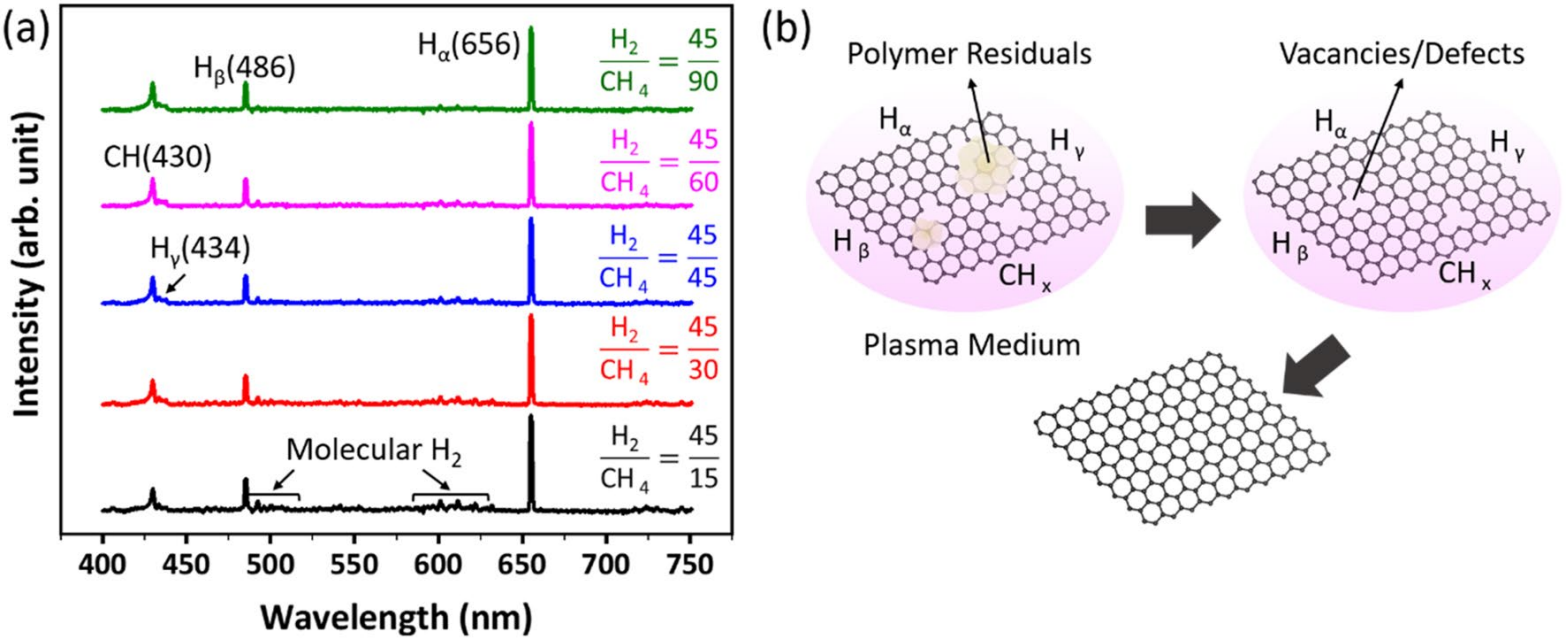
(**a**) Spectra of optical emission spectroscopy of methane + hydrogen plasma for each gas ratio value, at a pressure of 80 mtorr with a power of 80 watts. (**b**) Schematic of recovery and enhancement of Gr in methane + hydrogen plasma.

Figure 1a indicates the OES of plasma from a mixture of hydrogen/methane gas under optimal conditions (power 80 W, pressure 80 mTorr, methane/hydrogen 30/45), including H_y_ and CH_x_ radiation. In the hydrogen spectrum, H_α_ line (H (n = 3 → *n* = 2)), H_β_ line (H (n = 4 → *n* = 2)) and H_γ_ line (H (n = 5 → *n* = 2)) are related to the Ballmer series of hydrogen in 656, 486 and 434 wavelengths, respectively. The CH radical emission peak is also seen at 430 nm. The presence of hydrogen affects the conversion of CH_x_ species to more active species. It prevents the recombination of methane molecules, significantly affecting the deposition process and eliminating graphene lattice defects. All possible excitation processes are listed below^35^:

$${CH}_{4}+e\to CH\left({A}^{2}\Delta \right)+{H}_{2}+H+e$$

$${CH}_{4}+e\to {CH}_{3}+H\left(n=4\right)+e$$

$${CH}_{4}+e\to {CH}_{2}+{H}_{2}\left({G}^{1}{\Sigma }_{g}^{+}\right)+e$$

$${H}_{2}+e\to H\left(n=4\right)+H+e$$

$$H+e\to H\left(n=4\right)+e$$

It should be noted that H alpha and H gamma emission lines follow almost the same procedure as the H beta emission line^35^.

Figure 1b shows a schematic of plasma processing. The active hydrogen species produced in plasma are potent etching agents that clear the polymer residuals and oxygen functional groups by physical and chemical reactions. As a result, the entire surface of graphene is wholly exposed to plasma; therefore, the defects that exist under the polymer residue or the others induced to the Gr lattice due to the removal of the impurities can be eliminated by the CH_4_ active species during the mechanism mentioned before.

To achieve the optimal graphene processing conditions, a large number of experiments were performed with different conditions of pressure, gas ratio, and plasma power. A comparison between these conditions and the amount of active species formed by spectroscopy can be found in Supplementary Information. When the amount of hydrogen was too high, we saw the graphene layers degradation and even its complete disappearance from the substrate. On the other side, we saw amorphous carbon deposition on the substrate and even the chamber's inner walls when the methane content was higher than the optimal value.

According to experiments, the optimal conditions for achieving high-quality graphene are established when the ratio of Ha to CH is numerically around 3.5. This ratio alone is not a determining parameter; at high powers, the ions in the plasma will be more energetic, exposure of energetic ions to graphene causes defects or even total damage of the layer. On the other hand, the creation of active species occurs less at low powers. Even active Hydrogen species do not have enough numbers and energy to etch the polymer. In addition, there is a lack of CH species to eliminate defects and vacancies in low power plasma. For the characterization of the induced changes, we use Raman spectroscopy. The Raman spectrum of Gr has two distinct bands, which are labeled as G (1580 cm^−1^) and 2D (2700 cm^−1^). In the presence of disorder in Gr lattice, we can also see D-band (1350 cm^−1^) in the spectrum at about half of the frequency of the 2D-band. The G-band is related to electron–phonon coupling interaction, and its position can change with the state of disorder in materials^36^. For our sample, before and after the plasma treatment, G-peak's position and intensity did not change.

**Figure 2:**
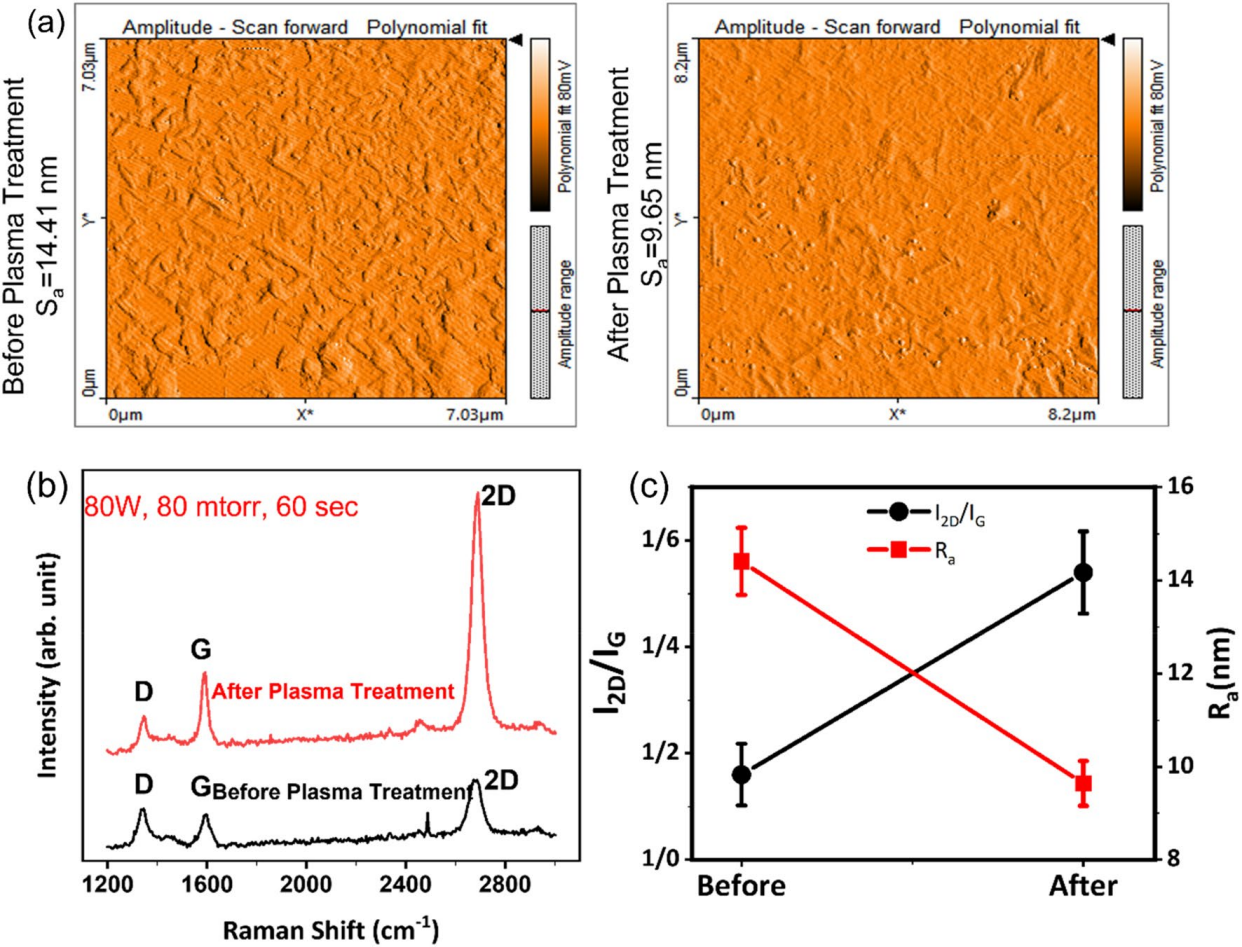
(**a**) AFM of Gr layer before (left image) and after (left image) plasma treatment. (**b**) Raman spectra of a transferred CVD Gr layers on SiO_2_ substrate before and after the plasma processing. (**c**) Gr surface roughness, and I_2D_/I_G_ value for the Gr before and after plasma processing (the line is plotted as a guide to the eye).

Figure 2a shows the AFM images of graphene before and after plasma treatment with optimized conditions. After plasma processing, graphene has a smoother surface with less roughness, which indicates the removal of polymer residuals on the surface of Gr by plasma etching. The D peak in Fig. 2b is related to the defects in the Gr. Environmental effects and also transferring of Gr through wet method can be the reason for these defects. As we can see in Fig. 2, the D peak intensity decreases after the plasma processing, which means that the sheets have fewer defects than before plasma processing. The quality is higher for the plasma-treated sample. By increasing the number of layers, the width of the 2D peak (2690 cm^−1^) gets wider^37^. The 2D peak in bulk graphite has two components, 2D_1_ and 2D_2_, and by decreasing the number of layers, it becomes sharp and forms single peak^38^. This is also evident in Fig. 2b for the sample after the plasma processing. Also, the I_2D_/I_G_ intensity ratio is a function of Gr layer thickness. For a single layer of Gr, this ratio is about 4.1 and for the bulk is about 0.31, so it means by increasing the number of the layers, this ratio gets smaller^39^. After plasma processing, the I_2D_/I_G_ intensity ratio increases, which means that some layers are lifted from the sample, and the Gr layer becomes thinner^40^. These changes are depicted in Fig. 2. Therefore, both the defect healing and polymer removal are resulted by using our plasma treatment. Further characterizations like high resolution transmission electron microscopy (HRTEM) are suggested to better reveal the role of plasma treatment on the healing of the defects in Gr. But according to the nature of the plasma gas and the chemical reactions presented in Fig. 1 we conclude that the changes in the Raman response and transport properties are also affected by the healing effects in the Gr layer.

**Figure 3:**
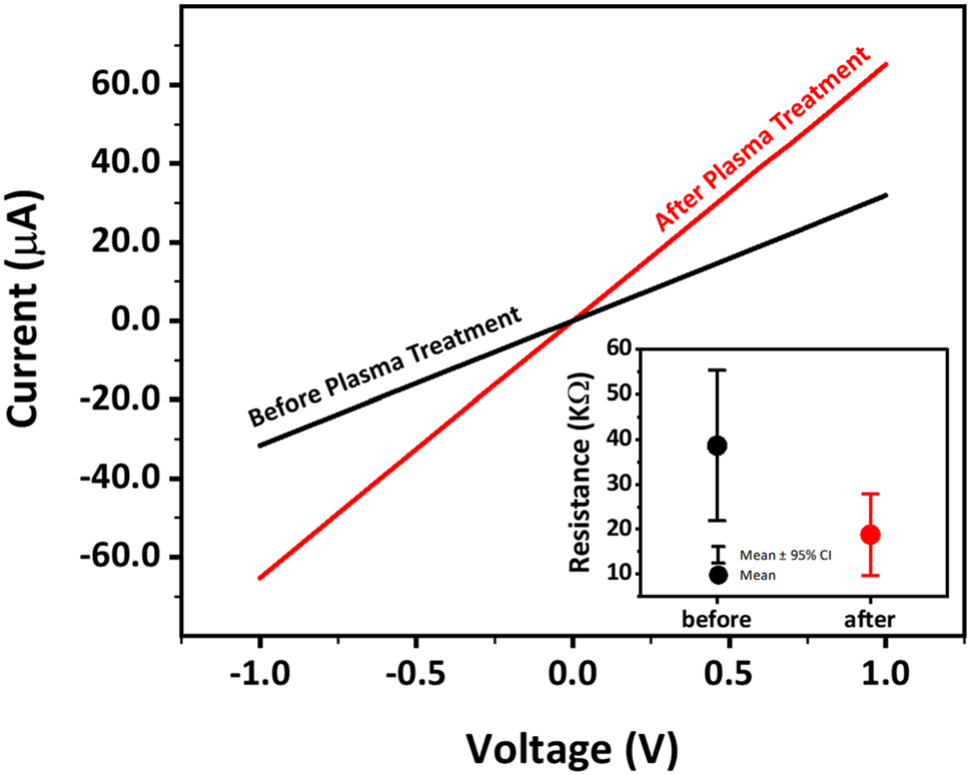
I–V curve of the Gr related to the layer before and after the plasma processing. The plasma treatments improved the electrical conductivity.

Finally, to see the effect of the polymer removal, defects healing, and thickness decrease on the transport properties, I–V measurements were performed. In Fig. 3, the I–V curve of graphene before and after plasma processing is shown. Gr with hydroxyl and carboxyl functional groups has a semiconductive property and can result in Schottky contact. In our sample before plasma treatment, the I–V curve briefly deviates from a completely linear behavior, which might be due to the presence of a small amount of hydroxyl and carboxyl functional group^41–43^. This nonlinearity in the I–V curve disappeared after plasma treatment, which might be due to the removal of the hydroxyl and carboxyl functional groups by plasma treatment. The inset of Fig. 3 shows the resistivity of the samples calculated from the slope of the I–V curve at the origin and also the probe distances. The error bar is obtained from several measurements performed for each sample. As can be seen, the resistivity of the Gr is decreased by plasma treatment. The electrical properties of graphene can be due to the scattering of charged impurities^41^. In-plane conductivity of Gr is significantly dependent on Gr structure and purity such that the presence of structural disorders, defects, and bonding functional groups such as oxygen groups reduces the electrical conductivity of Gr^44^. Vacancies that are scattering centers^45^ and defects reduce the mobility of charge carriers that cause increasing the Gr resistivity^46,47^. Also, conductivity is inversely proportional to the number of layers, so that single-layer graphene has the highest conductivity^48^. It is known that the sample conductivity increases after the healing process and is related to the recovery of sp^2^ C=C bonds (graphitization) in graphitic structures^49^. In our sample, both the thickness change and defects healing have resulted in the observed increase of conductivity, and we cannot separate their contributions.

## Conclusions

In summary, we obtained the optimal plasma conditions (pressure, power, and gas ratio) of a mixture of methane and hydrogen plasma by OES to balance the etching and deposition rate and reduce the defects and disorder of Gr, and also remove the polymer residuals properly. These were evidenced by comparing the Raman and AFM measurements before and after plasma treatment. Also, we showed that by plasma treatment, the electrical conductivity increased by ~ 100% and can result from defects healing and thickness change. Results are important towards improving Gr quality for using in electronic devices (Supplementary Information).

## Supplementary Information

Supplementary Information.

## Data Availability

The data that support the findings of this study are available from the corresponding authors upon reasonable request.
