# Supplementary material for: Low defect and high electrical conductivity of graphene through plasma graphene healing treatment monitored with in situ optical emission spectroscopy
Source: Sci Rep. 2021 Oct 13;11:20334. doi: 10.1038/s41598-021-99421-7 (PMC8514466; doi:10.1038/s41598-021-99421-7)
Supplement: Supplementary file 1 — Supplementary Information. [file 41598_2021_99421_MOESM1_ESM.pdf]

Supporting Information for

Low defect and high electrical conductivity of graphene through plasma graphene healing treatment monitored with in situ optical emission spectroscopy

Mohammad Salehi[1], Parnia Bastani[2], Loghman Jamilpanah[2], Abbas Madani[3,4]
Seyed Majid Mohseni[2,*], Babak Shokri[1,2,**]

[1]Laser and Plasma Research Institute, Shahid Beheshti University, Tehran 19839, Iran
[2]Department of Physics, Shahid Beheshti University, Tehran 19839, Iran
[3]AMO GmbH (Advanced Microelectronic Center), Aachen, Germany
[4] Department of Engineering, The University of Cambridge, Cambridge UK

[**]corresponding author: Babak Shokri (b-shokri@sbu.ac.ir)
[*]co-corresponding author: Seyed Majid Mohseni (m-mohseni@sbu.ac.ir)

1- Plasma Setup

In this study, a steel chamber was used in which the capacitor coupled plasma is generated by applying a voltage through an R.F. (13.56 MHz) power supply. This steel chamber has a diameter of 30 cm and a height of 40 cm and includes R.F. inlet ports, water inlet pipes for cooling the cathode electrode, and gas inlet ports installed in the lower part the chamber. There are Two electrodes in this chamber, to which one of them is connected to R.F. power. This electrode has a diameter of 15 cm. On this electrode, a negative bias voltage is induced. As this voltage increases, the energy of the ion bombardment surface increases so we could tune the ion energies for better results. The chamber body is grounded, with four other inlets for measuring the pressure inside the chamber and other plasma parameters and quartz windows for optical examinations on either side of the chamber.

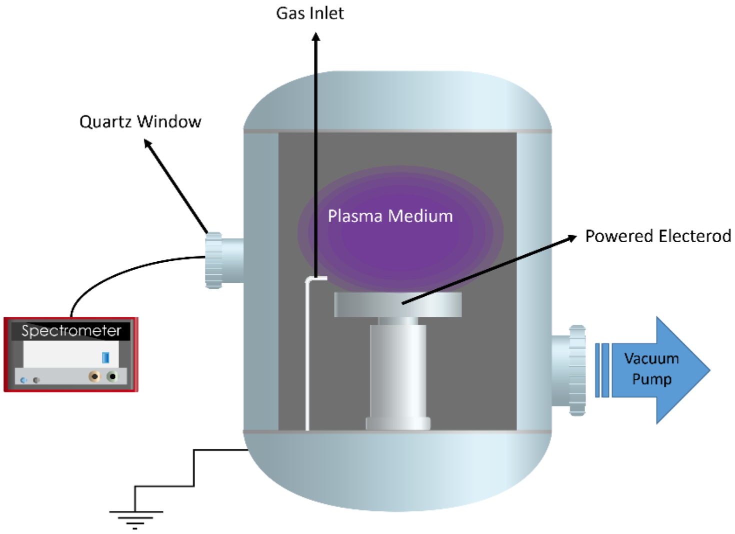

Figure 1 Schematic of vacuum chamber and spectrometer

2- Plasma Spectroscopy

Plasma can separate methane molecules and form CH_x species ($x < 4$). The generated CH_x species are so active that they can produce graphene at temperatures lower than normal graphene growth temperatures. The activation energy for direct growth of graphene on a dielectric substrate is 1.03 eV using 50 watts of R.F. plasma versus 2 - 2.6 eV with thermal CVD. Pure CH_4 plasma is likely to lead to the formation of amorphous carbon sp3, which inhibits the growth of graphene crystals. Plasma-produced H species can etch the edges of graphene and prevent the formation of graphene nanoclusters, thus creating the conditions for the growth of new graphene. Hydrogen plasma plays an essential role in controlling the structure, domains, and layers and removing oxidizing agents.[1–7]

Optical Emission Spectroscopy (OES) with 0.6 nm spectral resolution (AvaSpec 3648, Avantes) was performed for different conditions such as gas ratio (methane/hydrogen [15/45, 30/45, 45/45, 45/60, 45/90]). Different powers (40, 80, 120 watts) and also pressures between 40 and 120 mtorr. To detect the various kinds of species and ionized atoms and molecules that emerge within the plasma. The diagrams in Figure 2 show the OES of the plasma under different test conditions. In all experiments, the active species produced are almost the same. However, as it is obvious in the tables, the density of each active species and the ratio of the densities of the active species are different, which means that in some experiments, the plasma behavior and the result of the plasma interaction with the sample are different.

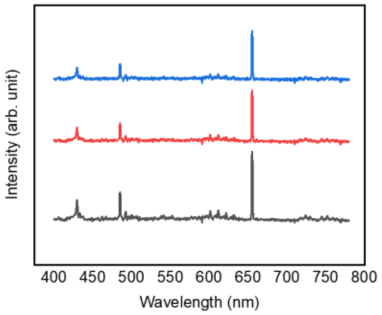

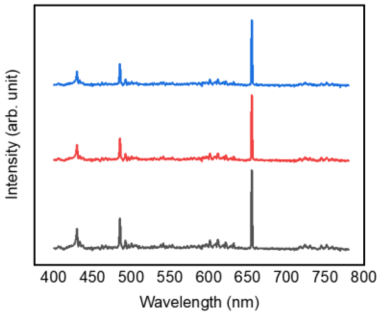

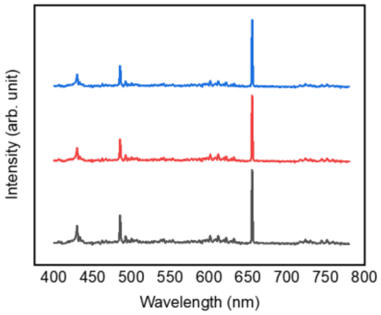

	Power = 40 Watts Gas Ratio = H_2/CH_4 - 45/15		
	**40 mtorr**	**80 mtorr**	**120 mtorr**
H_α/H_β	2.384025	2.769015	2.98871
H_α/CH	3.231339	3.468777	3.918928
H_β/CH	1.355413	1.252712	1.311244

	Power = 80 Watts Gas Ratio = H_2/CH_4 - 45/15		
	**40 mtorr**	**80 mtorr**	**120 mtorr**
H_α/H_β	2.493828	2.88236	2.961714
H_α/CH	3.692995	4.046656	4.506163
H_β/CH	1.480854	1.403938	1.521471

	Power = 120 Watts Gas Ratio = H_2/CH_4 - 45/15		
	**40 mtorr**	**80 mtorr**	**120 mtorr**
H_α/H_β	2.477352	2.872594	3.109438
H_α/CH	3.897533	4.626367	5.221708
H_β/CH	1.573266	1.610519	1.679309

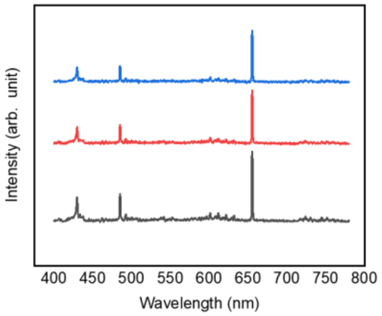

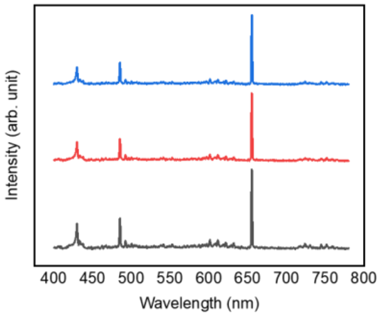

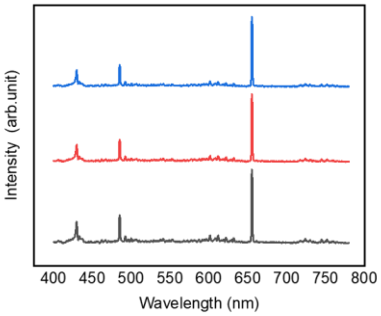

	Power = 40 Watts Gas Ratio = H_2/CH_4 - 45/30		
	**40 mtorr**	**80 mtorr**	**120 mtorr**
H_α/H_β	2.377841	2.577164	2.92352
H_α/CH	2.705703	3.078792	3.134722
H_β/CH	1.137882	1.194643	1.072242

	Power = 80 Watts Gas Ratio = H_2/CH_4 - 45/30		
	**40 mtorr**	**80 mtorr**	**120 mtorr**
H_α/H_β	2.549179	2.999439	3.160668
H_α/CH	3.064359	3.535283	3.928776
H_β/CH	1.202097	1.178648	1.243021

	Power = 120 Watts Gas Ratio = H_2/CH_4 - 45/30		
	**40 mtorr**	**80 mtorr**	**120 mtorr**
H_α/H_β	2.570223	3.001444	3.278705
H_α/CH	3.300948	3.874239	4.112615
H_β/CH	1.284304	1.290792	1.254341

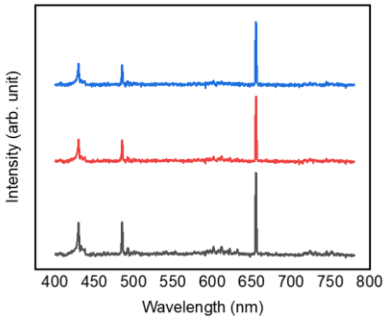

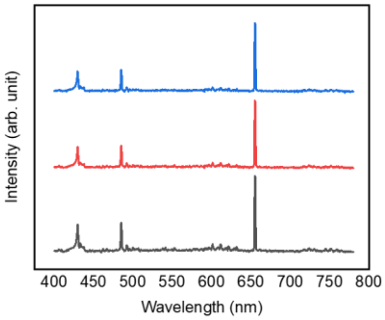

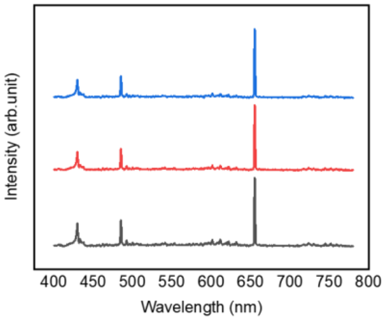

	Power = 40 Watts Gas Ratio = H_2/CH_4 - 45/45		
	40 mtorr	80 mtorr	120 mtorr
H_α/H_β	2.412834	2.967231	3.018782
H_α/CH	2.445734	2.828979	2.831770
H_β/CH	1.013635	0.953406	0.938050

	Power = 80 Watts Gas Ratio = H_2/CH_4 - 45/45		
	40 mtorr	80 mtorr	120 mtorr
H_α/H_β	2.548668	2.951351	3.080724
H_α/CH	2.721887	3.119518	3.338201
H_β/CH	1.067964	1.056979	1.083576

	Power = 120 Watts Gas Ratio = H_2/CH_4 - 45/45		
	40 mtorr	80 mtorr	120 mtorr
H_α/H_β	2.548572	2.998380	3.130369
H_α/CH	2.858378	3.40466	3.734752
H_β/CH	1.121560	1.135501	1.193071

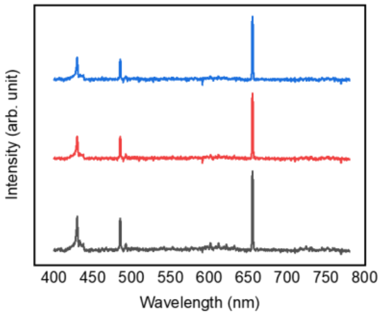

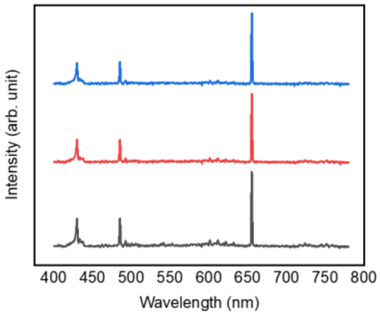

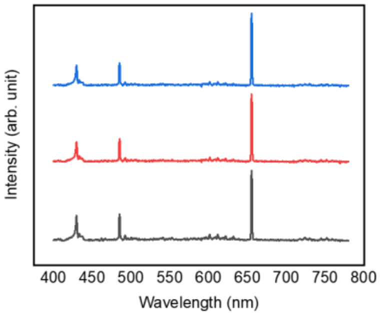

	Power = 40 Watts Gas Ratio = H_2/CH_4 - 45/60		
	40 mtorr	80 mtorr	120 mtorr
H_α/H_β	2.392575	2.891311	3.218976
H_α/CH	2.262871	2.856544	2.783139
H_β/CH	0.945789	0.987975	0.864604

	Power = 80 Watts Gas Ratio = H_2/CH_4 - 45/60		
	40 mtorr	80 mtorr	120 mtorr
H_α/H_β	2.537264	2.987871	3.165252
H_α/CH	2.53338	2.960386	3.304978
H_β/CH	0.998469	0.990801	1.044144

	Power = 120 Watts Gas Ratio = H_2/CH_4 - 45/60		
	40 mtorr	80 mtorr	120 mtorr
H_α/H_β	2.563479	2.969853	3.114959
H_α/CH	2.686225	3.353071	3.417763
H_β/CH	1.047882	1.129036	1.097210

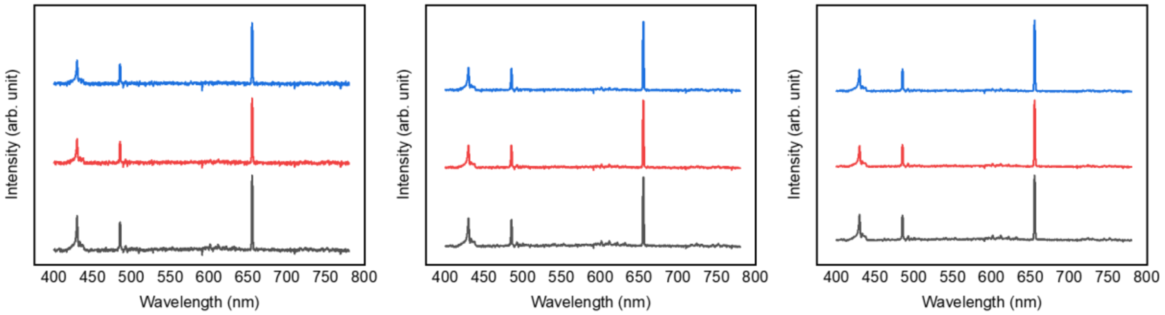

	**Power = 40 Watts Gas Ratio = H_2/CH_4 - 45/90**		
	**40 mtorr**	**80 mtorr**	**120 mtorr**
H_α/H_β	**2.518136**	**2.872549**	**2.994534**
H_α/CH	**2.071054**	**2.537795**	**2.540371**
H_β/CH	**0.822455**	**0.883465**	**0.848336**

	**Power = 80 Watts Gas Ratio = H_2/CH_4 - 45/90**		
	**40 mtorr**	**80 mtorr**	**120 mtorr**
H_α/H_β	**2.539734**	**2.909810**	**3.116210**
H_α/CH	**2.397586**	**2.953023**	**3.014901**
H_β/CH	**0.944030**	**1.014851**	**0.967489**

	**Power = 120 Watts Gas Ratio = H_2/CH_4 - 45/90**		
	**40 mtorr**	**80 mtorr**	**120 mtorr**
H_α/H_β	**2.519662**	**2.932915**	**3.125715**
H_α/CH	**2.453830**	**3.065748**	**3.173899**
H_β/CH	**0.973873**	**1.045290**	**1.015415**

Figure 2 Diagrams of active species generated in plasma under different conditions

References

1. Liu, D. *et al.* Two-step growth of graphene with separate controlling nucleation and edge growth directly on SiO2 substrates. *Carbon N. Y.* **72**, 387–392 (2014).

2. Sun, J., Zhang, Y. & Liu, Z. Direct chemical vapor deposition growth of graphene on insulating substrates. *ChemNanoMat* **2**, 9–18 (2016).

3. Wei, D. *et al.* Critical Crystal Growth of Graphene on Dielectric Substrates at Low Temperature for Electronic Devices. *Angew. Chemie* **125**, 14371–14376 (2013).

4. Van Der Laan, T., Kumar, S. & Ostrikov, K. K. Water-mediated and instantaneous transfer of graphene grown at 220 °c enabled by a plasma. *Nanoscale* **7**, 20564–20570 (2015).

5. Xie, L., Jiao, L. & Dai, H. Selective etching of graphene edges by hydrogen plasma. *J. Am. Chem. Soc.* **132**, 14751–14753 (2010).

6. Kim, Y. S. *et al.* Direct integration of polycrystalline graphene into light emitting diodes by plasma-assisted metal-catalyst-free synthesis. *ACS Nano* **8**, 2230–2236 (2014).

7. Wei, D. *et al.* Low temperature critical growth of high quality nitrogen doped graphene on dielectrics by plasma-enhanced chemical vapor deposition. *ACS Nano* **9**, 164–171 (2015).
